# Global Health Research Mentoring Competencies for Individuals and Institutions in Low- and Middle-Income Countries

**DOI:** 10.4269/ajtmh.18-0558

**Published:** 2018-11-14

**Authors:** Davidson H. Hamer, Bhakti Hansoti, Dorairaj Prabhakaran, Mark D. Huffman, Nonhlanhla Nxumalo, Matthew P. Fox, Satish Gopal, Richard Oberhelman, Lawrence Mwananyanda, Bellington Vwalika, Laetitia C. Rispel

**Affiliations:** 1 Department of Global Health, Boston University School of Public Health, Boston, Massachusetts;; 2 Section of Infectious Diseases, Department of Medicine, Boston Medical Center, Boston, Massachusetts;; 3 Department of Emergency Medicine, Johns Hopkins School of Medicine, Baltimore, Maryland;; 4 Department of International Health, Johns Hopkins School of Public Health, Baltimore, Maryland;; 5 Centre for Control of Chronic Disease, Public Health Foundation of India, Delhi, India;; 6 London School of Hygiene and Tropical Medicine, London, United Kingdom;; 7 Department of Preventive Medicine, Northwestern University Feinberg School of Medicine, Chicago, Illinois;; 8 Faculty of Health Sciences, Centre for Health Policy, School of Public Health, University of the Witwatersrand, Johannesburg, South Africa;; 9 Department of Epidemiology, Boston University School of Public Health, Boston, Massachusetts;; 10 Health Economics and Epidemiology Research Office, Department of Internal Medicine, School of Clinical Medicine, Faculty of Health Sciences, University of the Witwatersrand, Johannesburg, South Africa;; 11 Departments of Medicine and Epidemiology, University of North Carolina, Chapel Hill, North Carolina;; 12 Department of Medicine, University of Malawi College of Medicine, Lilongwe, Malawi;; 13 Department of Global Community Health and Behavioral Sciences, Tulane University School of Public Health and Tropical Medicine, New Orleans, Louisiana;; 14 Right to Care Zambia, Lusaka, Zambia;; 15 University of Zambia School of Medicine, Lusaka, Zambia;; 16 Department of Science and Technology/ National Research Foundation Research Chair, School of Public Health, University of the Witwatersrand, Johannesburg, South Africa

## Abstract

Mentoring is beneficial to mentors, mentees, and their institutions, especially in low- and middle-income countries (LMICs), that are faced with complex disease burdens, skills shortages, and resource constraints. Mentoring in global health research can be enhanced by defining key competencies, to enable the skill set required for effective mentoring, determine training needs for local research mentors, and facilitate institutional capacity building to support mentors. The latter includes advocating for resources, institutional development of mentoring guidelines, and financial and administrative support for mentoring. Nine core global health research mentoring competencies were identified: maintaining effective communication; aligning expectations with reasonable goals and objectives; assessing and providing skills and knowledge for success; addressing diversity; fostering independence; promoting professional development; promoting professional integrity and ethical conduct; overcoming resource limitations; and fostering institutional change. The competencies described in this article will assist mentors to sharpen their cognitive skills, acquire or generate new knowledge, and enhance professional and personal growth and job satisfaction. Similarly, the proposed competencies will enhance the knowledge and skills of mentees, who can continue and extend the work of their mentors, and advance knowledge for the benefit of the health of populations in LMICs.

## Introduction

High-quality mentorship has the ability to transform the trajectory of individual career paths and to shape the identity and success of institutions.^1^ Effective mentorship helps mentees to reach their full potential, create and disseminate new knowledge, invoke positive institutional change, and build local capacity. The benefits of effective mentorship extend into the health-care system and can lead to improved quality of clinical care in resource-limited settings.^2^

The characteristics and skills required for effective mentorship have been described previously and include the capacity to communicate with empathy, compassion, and respect; integrity; and the ability to establish and maintain a trusting relationship.^3–6^ Research mentors should have technical expertise in all aspects of the responsible conduct of research, including grant proposal writing, study design, data collection, analysis, the dissemination of results, and policy engagement to ensure that the results are translated into policy or practice. Newer trends in research mentorship take a more holistic view, with mentors not only supporting development in research-focused endeavors but also in leadership, wellness, and personal growth. While a general understanding of the attributes of a successful mentor is known, there is limited information on the competencies required for effective global health research mentorship, especially in low- and middle-income countries (LMICs).^7,8^ These countries share common challenges of complex disease burdens, skill shortages, underinvestment in research, and resource constraints.^9^

In a LMIC context, the mentor, whether this individual is from a LMIC or based in a high-income country (HIC) and collaborates in a LMIC, must be comfortable with cross-cultural communication, an ability to overcome limited or inconsistent institutional support, and capacity to provide effective mentorship in the context of limited infrastructure and financial support. Mentors should thus be ready to provide flexible and diverse skills including lifelong learning, necessary for career success.

Another challenge in the LMIC context is the lack of or shortages of skilled mentors, able to guide mentees holistically. A single mentor may need to fulfill multiple roles (i.e., simultaneously play the role of research, career development, and personal mentor) for numerous mentees and will have to take on a wider breadth of roles and responsibilities (such as early leadership and mentoring roles). By contrast, in HICs such as the United States, an increasingly common model is the development of mentorship teams, which when combined, fulfills the numerous roles required in the holistic mentorship model. In some LMICs, having a team of mentors with different levels of expertise or different mentorship roles from two or more institutions is an increasingly common model for research.

Competencies are defined as specific skills, techniques, attitudes, and knowledge that mentors can develop through training, education, and experience.^10–12^ Mentoring competencies include knowledge and skill in the structure and process of the mentoring relationship, ability to cope with the challenges arising with a mentoring relationship, and an understanding of the roles and responsibilities of the mentoring relationship. Defining competencies for mentoring in a global health context is important for several reasons: providing an opportunity to define the skill set required for effective mentoring in LMICs; determining the training needs for local research mentors;^13–16^ and advocating for resources, institutional development of guidelines, and support for mentoring for basic, clinical, and/or public health research.^17^ This article proposes a framework of competencies required for mentoring in a global health research context for mentors and institutions in LMICs.

## Approach

We adapted two major sources describing research mentoring competencies that were developed primarily for use in mentoring in HICs.^7,10^ These were augmented by the outcomes of four mentorship training workshops held in Africa, Asia, and South America^18^; summary discussions from a global health research mentoring workshop held in Virginia in 2017; input from global health mentoring experts at a subsequent meeting in New York City in 2018; and a modified Delphi approach based on input from the coauthors. This combination of published descriptions of mentoring competencies and skills was supplemented with expert opinion to develop a table summarizing the key competencies necessary for research mentoring within a global health context in LMIC institutions.

## Global health research mentoring competencies

Although the proposed competencies may be applicable in HICs, north–south partnerships are at the heart of global health research initiatives. The proposed competencies will assist with more equitable relationships among researchers from different country contexts. Nine core competencies were identified, and six competencies were adopted by the work from Fleming et al.^8^ These include 1) maintaining effective communication, 2) aligning expectations, 3) providing skills and knowledge for success, 4) addressing diversity, 5) fostering independence, and 6) promoting professional development. To expand beyond a pure research paradigm and taking into account the particular challenges faced by mentors in a LMIC setting, three additional competencies have been added: promoting professional integrity and ethical conduct, overcoming resource limitations, and fostering institutional change. Table 1 builds on these nine core competencies and identifies the supporting skills required to attain proficiency and provides specific examples of how they can be measured in an LMIC context.

**Table 1:** An adapted framework for global health research mentoring competencies and skills

Competency	Skills, attitudes, and knowledge	Potential milestones/performance measures
Maintaining effective communication	Listening with the intention to understand mentees issues and concerns	Mentee evaluation forms/feedback
	Demonstrating interest/attention	
		Self-reporting
	Encouraging mentees to speak	
		Peer evaluationFrequency of mentoring meetings
	Openness to visual and other nonverbal signals	
	Availability to meet on a regular basis	
	Demonstrate skill and sensitivity with cross-cultural and/or cross-gender communication	
	Able to communicate with empathy and compassion	
	Coordinate or work collaboratively with other mentors	
	Provide constructive feedback for written and oral work	
Aligning expectations with reasonable goals and objectives	Demonstrate altruism and enthusiasm about mentoring	Self-reporting
	Practice reflexivity and demonstrate self-awareness	
		Objective measures on mentee progress
	Assist with research goal setting and realistic project planning	
	Help mentee to develop strategies to attain goals	
		Quantity and scope of mentees
	Assist mentee to implement strategies and plans	
Assessing and providing skills and knowledge necessary for success	Knowledge of relevant subject matter (helpful but not obligatory)	Monitors own accomplishments in terms of clinical status, publications, and secured grant funding
	Competence in all aspects of research from proposal writing to dissemination and policy engagement	
	Able to identify key gaps in knowledge and skills of the mentoring team	
	Proactively assist with recruiting co-mentors to fill these areas of need or refer mentee for expert advice (when needed)	
Addressing diversity	Demonstrate willingness for ongoing learning or training in critical diversity	Provides mentoring to a diverse group regardless of gender, culture, and race/ethnicity
	Embrace diversity by accepting and encouraging collaboration with individuals, regardless of gender, culture, nationality, race/ethnicity, sexual orientation, and/or religious backgrounds	
		Training/reading in critical diversity
	Ability to recognize own biases, whether conscious or unconscious, and willingness to engage or work to overcome these	
Fostering independence	Help establish research career goals and motivate mentee to meet those goals	Mentee’s success in securing independent funding
	Assist with identification of potential funding opportunities	
		Promotion of mentees to institutional, clinical, or academic leadership roles
	Demonstrate belief in mentee(s)	
	Help mentee to build or enhance self-confidence	
	Provide networking opportunities	
		Evaluation/feedback from mentee
	Willing to step back to a supporting role	
	Help mentee to take increasing responsibilities for managing the relationship	
	Allow successful mentees to function as group leaders when appropriate	
Promoting professional development	Willing to invest time to foster development of mentees	Creation of individual development plan (IDP)
	Support (financially/time) a plan to attain career goalsHelp mentee to develop effective and broad communication skills (e.g., public speaking and writing)Guide mentee on working in a teamHelps mentee to balance work and personal lifeHelp mentee to prioritize and manage timeAssist mentee to redefine the mentoring the relationship	
		Mentee satisfaction scoresProgress of mentee’s research and professional career
Promoting professional integrity and ethical conduct	Demonstrate honesty and openness with mentee	Responsible conduct of researchBalance authorship on manuscripts with menteesMentee feedback
	Serve as a role model in terms of personal and work behaviors	
	Provide guidance on responsible conduct of research—both approvals and implementation	

	Able to develop a trusting relationship	
	Acknowledge contributions of the mentee	
	Raise awareness about importance of ethical guidelines and institutional rules	

	Demonstrate commitment to own learning and interest in helping others to learn	
Overcoming resource limitations and other sources of adversity	Provide training on managing administrative duties	Development of local leadership training programsMentee success in obtaining research funding
	Demonstrate and teach problem-solving skills and patience in the face of setbacks	
	Help to identify nonfinancial and financial resources needed to accomplish research goals	

	Guide mentee to identify existing institutional resources and how to optimize these	
	Assist mentee to develop solutions to overcome barriers to research (e.g., internal politics and local bureaucracy)	
Fostering institutional change	Advocate for institutional support for mentoring and both mentor and mentee	Funding, equipment, and other resources secured for the mentee
	Advocate for resources (space, equipment, and support staff) to build local capacity	
		Institutional mentoring environment

## Discussion

Communication competency addresses the interpersonal communication capacity and the self-awareness of the mentor that make transfer of knowledge and skills possible,^10^ and serves as one of the core competencies associated with effective mentoring.^19^ Extra attention should be paid to communication skills that facilitate cross-cultural and cross-gender mentoring in a LMIC setting. This is especially important because many academic institutions in LMICs are dominated by men, and a lack of appreciation of the importance of gender and ethnic diversity for broad-based success has been identified as a barrier to effective mentoring.^20,21^ We suggest that institutions in LMICs should make special efforts to encourage the growth of female researchers. This includes supporting female role models to mentor young women as global health researchers, improving the visibility and acknowledgment of female academics and funding schemes that prioritize the research careers of women, by having a higher age cutoff for female grant recipients. Behaviors that foster women’s growth and development include empathy and caring, recognizing the multiple roles of women in patriarchal societies, and adjusting the work environment where possible.

One of the coauthors (Laetitia C. Rispel) has substantial experience mentoring young women researchers of color successfully. In qualitative interviews conducted in 2017, female mentees were asked to describe factors that facilitated their professional and personal growth.^22^ The respondents identified five central themes in regard to mentorship of young women scientists including 1) the importance of leadership and role modeling by mentors; 2) putting women first in a practical sense, for example, as a first author in an article, or making time for a mentoring session, despite competing priorities; 3) creating an enabling and supportive environment, by recognizing the multiple roles and responsibilities of young women and supporting them both personally and professionally; 4) identification of potential, and instilling confidence and self-belief; and 5) encouraging reflexive practice and reminding the mentees to take care of themselves. These concepts can be successfully integrated into mentorship training programs and are aligned with the core competencies discussed in this article.

Competencies surrounding aligning expectations, and providing skills and knowledge for success, require a competent mentor to stimulate academic output (e.g., grant proposals, research presentations, abstracts, and manuscripts), and an ability of the mentor to assess the mentee’s current knowledge level and develop joint strategies and tactics to attain mentee’s goals. Given the relative scarcity of mentors in LMICs and the resulting need for a broad mentorship portfolio for many mentors, scientific networks can be used to find additional mentors as needed and should maximize south-to-south and south–north collaborations in global health. In addition, self-awareness, curiosity, reflexivity, and demonstrating a willingness to learn should be cultivated among mentors and mentees.

Addressing diversity necessitates a critical approach that requires the mentor to recognize the value of hegemonic identities (e.g., white privilege, heterosexuality, masculinity, and institutional hegemony); be able to analyze how systems of racial, gender, or class oppression intersect with one other; and willingness to engage and work actively to transform oppressive systems.^23^

Professional development competencies focus on helping the mentee to develop and answer hypothesis-driven research questions that will allow mentees to become independent from their mentor over time. In addition, mentors need to help create or facilitate opportunities for networking for the mentee, and, once new collaborations have been established, to nurture the collaborations so that they will lead to high quality research output while concurrently allowing the mentee to gain new expertise. Assisting with professional development often includes providing career guidance, helping the mentee to work in teams or to balance work and their personal life, and, at times, helping the mentee work through personal and health issues that may arise. Throughout, the mentor must serve as a role model of professionalism. Competency surrounding independence also should focus on the mentor’s readiness to help their mentee with identifying potential sources for funding and to learn how to work with different funding agencies. This process may also require providing the mentee with insight into scientific, operational, and strategic aspects of research. Because many research projects in LMICs involve national and international collaborators and collaborating institutions, mentors need to be able to help mentees learn how to work effectively with and benefit from these collaborations.

Professional integrity and ethical competency are critical to help guide the mentee with ethical conduct of research, including but not limited to creation of ethically sound research protocols and projects, engagement of the community under study throughout the research process, informed consent of research participants, and understanding and adhering to standards and criteria for authorship as well as the importance of different authorship positions.^24^ The mentor should be willing to provide the mentee with opportunities to serve as the lead author on their research that they are conducting or lead a clinical working group that is implementing a new protocol. These concepts are more fully explored in a subsequent article in this special journal issue.^25^

The institutional commitment to mentorship may come in the form of policy statements, advancement and promotion, financial support, and the allocation of space and personnel.^26^ The competency of fostering institutional change is not only centered around advocacy but also requires the mentor to understand what is required at the institutional level to design and implement effective mentorship programs, and the ability to take on the responsibility to execute such programs.

The local institutional culture may play an important role in helping mentors develop the aforementioned competencies and ideally should recognize the importance of effective mentoring. The need for training mentors and providing them with financial and logistical support was identified in a survey of 22 mentors at Makerere University in Uganda.^17^ Training mentors may require making resources available for the development of internal curricula or funding participation in external international mentoring training opportunities such as the regional mentoring workshops organized by HIC and LMIC institutions funded by the Fogarty International Center and other organizations that focus on strengthening research capacity of investigators in LMICs. These regional workshops designed for leaders at LMIC institutions offer excellent opportunities for the participants to return home with training materials for subsequent adaptation and development of their own training workshops at their home institutions.

In settings where there is a limited number of available mentors,^17,27^ one potential solution is to use a peer mentoring strategy. Engaging and training peer leaders in a structured peer mentoring program has been shown to be an effective approach to mentor scarcity in Kampala, Uganda.^27^ However, this strategy also has major limitations because inexperienced peer mentors may provide incorrect or conflicting advice.

## Conclusion

Mentoring is mutually beneficial to mentors and mentees, as well as their institutions, and it is critical to mentee development to create the next generation of research leaders. More empirical research is needed in LMICs that will demonstrate the benefit of formal mentoring programs and their return on investment. Nevertheless, the mentoring competencies listed herein will assist mentors and institutions to stay up to date with global health knowledge, sharpen their cognitive skills, and enhance both professional and personal growth and job satisfaction. Similarly, the proposed competencies will enhance the skills and experience of mentees and encourage them to generate or advance knowledge for the benefit of the health of populations in LMICs.
